# Deep ocean drivers better explain habitat preferences of sperm whales *Physeter macrocephalus* than beaked whales in the Bay of Biscay

**DOI:** 10.1038/s41598-022-13546-x

**Published:** 2022-06-10

**Authors:** Auriane Virgili, Valentin Teillard, Ghislain Dorémus, Timothy E. Dunn, Sophie Laran, Mark Lewis, Maite Louzao, José Martínez-Cedeira, Emeline Pettex, Leire Ruiz, Camilo Saavedra, M. Begoña Santos, Olivier Van Canneyt, José Antonio Vázquez Bonales, Vincent Ridoux

**Affiliations:** 1 Observatoire Pelagis, UAR 3462 CNRS - La Rochelle Université, 5 Allée de l’Océan, 17000 La Rochelle, France; 2 Joint Nature Conservation Committee, Inverdee House, Baxter Street, Aberdeen, AB11 9QA UK; 3 AZTI, Marine Research, Basque Research and Technology Alliance (BRTA), Herrera Kaia, Portualdea z/g, 20110 Pasaia, Spain; 4 CEMMA, Camiño Do Ceán, no 2, Nigrán, 36350 Pontevedra, Spain; 5 Cohabys - ADERA, La Rochelle Université, 2 rue Olympe de Gouges, Bâtiment ILE, 17000 La Rochelle, France; 6 ADERA, 162 avenue Albert Schweitzer, CS 60040, 33608 Pessac Cedex, France; 7 AMBAR Elkartea Organisation, Ondarreta Ibilbidea z/g, 48620 Plentzia, Bizkaia Spain; 8 Instituto Español de Oceanografía, Centro Oceanográfico de Vigo, 36390 Vigo, Spain; 9 Alnilam Research and Conservation, Pradillos 29, Navacerrada, 28491 Madrid, Spain; 10 Centre d’Etudes Biologiques de Chizé - La Rochelle, UMR 7372 CNRS - La Rochelle Université, Villiers-en-Bois, France

**Keywords:** Ecology, Conservation biology, Ecological modelling

## Abstract

Species Distribution Models are commonly used with surface dynamic environmental variables as proxies for prey distribution to characterise marine top predator habitats. For oceanic species that spend lot of time at depth, surface variables might not be relevant to predict deep-dwelling prey distributions. We hypothesised that descriptors of deep-water layers would better predict the deep-diving cetacean distributions than surface variables. We combined static variables and dynamic variables integrated over different depth classes of the water column into Generalised Additive Models to predict the distribution of sperm whales *Physeter macrocephalus* and beaked whales *Ziphiidae* in the Bay of Biscay, eastern North Atlantic. We identified which variables best predicted their distribution. Although the highest densities of both taxa were predicted near the continental slope and canyons, the most important variables for beaked whales appeared to be static variables and surface to subsurface dynamic variables, while for sperm whales only surface and deep-water variables were selected. This could suggest differences in foraging strategies and in the prey targeted between the two taxa. Increasing the use of variables describing the deep-water layers would provide a better understanding of the oceanic species distribution and better assist in the planning of human activities in these habitats.

## Introduction

The use of Species Distribution Models (SDMs), that allow to model the distribution of species by establishing relationships between species occurrence or abundance and environmental data^1^, has increased considerably in recent decades. They are commonly used to address ecological issues as well as for conservation purposes, *e.g.* to identify areas of concentration and inform policies for the designation of protected areas^2–4^. SDMs have been extensively applied to marine top predators (*e.g.*^5–8^), as these species are threatened by multiple anthropogenic activities (*e.g.* collisions, pollutants, bycatch, underwater noise, habitat loss) leading to the decline of many populations^9–11^.

The distribution of marine top predators is expected to be mainly driven by prey abundance or availability^12–15^; yet the limited spatial and temporal extent of prey data available from in situ sampling is a major impediment to model predator distributions over large oceanic regions. Most studies solve this lack of prey data by using static bathymetric variables depicting the seafloor together with surface dynamic environmental variables depicting the water masses, as proxies for prey distribution. Surface dynamic variables include *inter alia* sea surface temperature, sea surface height, sea surface currents, surface chlorophyll *a* concentration irrespective of the actual depth range used by the species of interest^5,8,16,17^.

These surface environmental data are easily accessible (obtained from satellite imagery or numerical models) and available at various spatial (local to global) and temporal scales and resolutions (*e.g.* day, month, year, decades)^6,18,19^. However, most prey targeted by marine top predators live in the water column at variable depths operating on diel migration patterns^20^. Using environmental variables depicting the water column would seem more relevant than using surface variables. This would be particularly true for oceanic species that spend most of their time in deep waters and generally feed on mesopelagic and bathypelagic prey like deep-diving cetaceans^21,22^, here referring to the beaked whales (family *Ziphiidae*, represented in the north Atlantic by *Ziphius cavirostris, Hyperoodon ampullatus* and *Mesoplodon* spp.) and the sperm whale (family *Physeteridae*, *Physeter macrocephalus*). Most studies that model the habitat of deep-diving cetaceans use static and dynamic surface variables (*e.g.*^5,16,23,24^). However, deep-divers regularly perform long dives, sometimes to great depths^25–27^ and consequently, surface variables may be partly irrelevant to predict the distribution of their deep-dwelling prey.

Brodie et al.^28^ included two subsurface dynamic variables (isothermal layer depth and bulk buoyancy frequency) in SDMs to describe the habitat of pelagic predators (swordfish *Xiphias gladius*, blue sharks *Prionace glauca*, common thresher sharks *Alopias vulpinus*, and shortfin mako sharks *Isurus oxyrinchus*) in the California Current System. The authors found these variables increased the explanatory power and predictive performance of the models for most species. Along the same lines, Becker et al.^29^ fitted SDMs to depth environmental variables provided by an ocean circulation model and to in situ and remotely sensed oceanic variables, both considered as measured data. They showed similar performance between the two models, highlighting the good performance of environmental modelled data to describe species distribution.

The development and increasing availability of environmental variables that describe the ocean in three dimensions opens the possibility of a better understanding of the distribution of species such as deep-diving cetaceans. Dynamic variables that characterise deeper oceanic layers are expected to have a greater influence on their prey fields at depth than surface parameters alone. We propose to test this hypothesis by combining static variables (that characterise seafloor topography) with dynamic variables integrated over different depth classes, such as temperature, gradients of temperature and eddy kinetic energy, in Generalised Additive Models (GAMs^30^;) to model the distribution of deep-diving cetaceans in the Bay of Biscay, eastern North Atlantic. GAMs have already demonstrated their effectiveness in predicting the distribution of deep-diving cetaceans^5,23,31^. In this study, several depth classes, corresponding to different environments in the water column, were defined and relevant environmental variables were extracted for each class. Then, GAMs were fitted to assess the explanatory power of these variables. Finally, the distribution of deep-divers in the Bay of Biscay was predicted using the best models obtained. We expected that the use of environmental variables at depth would result in a more accurate modelling of top predator densities and a better understanding of the mechanisms that influence their distributions.

## Results

**Figure 1:**
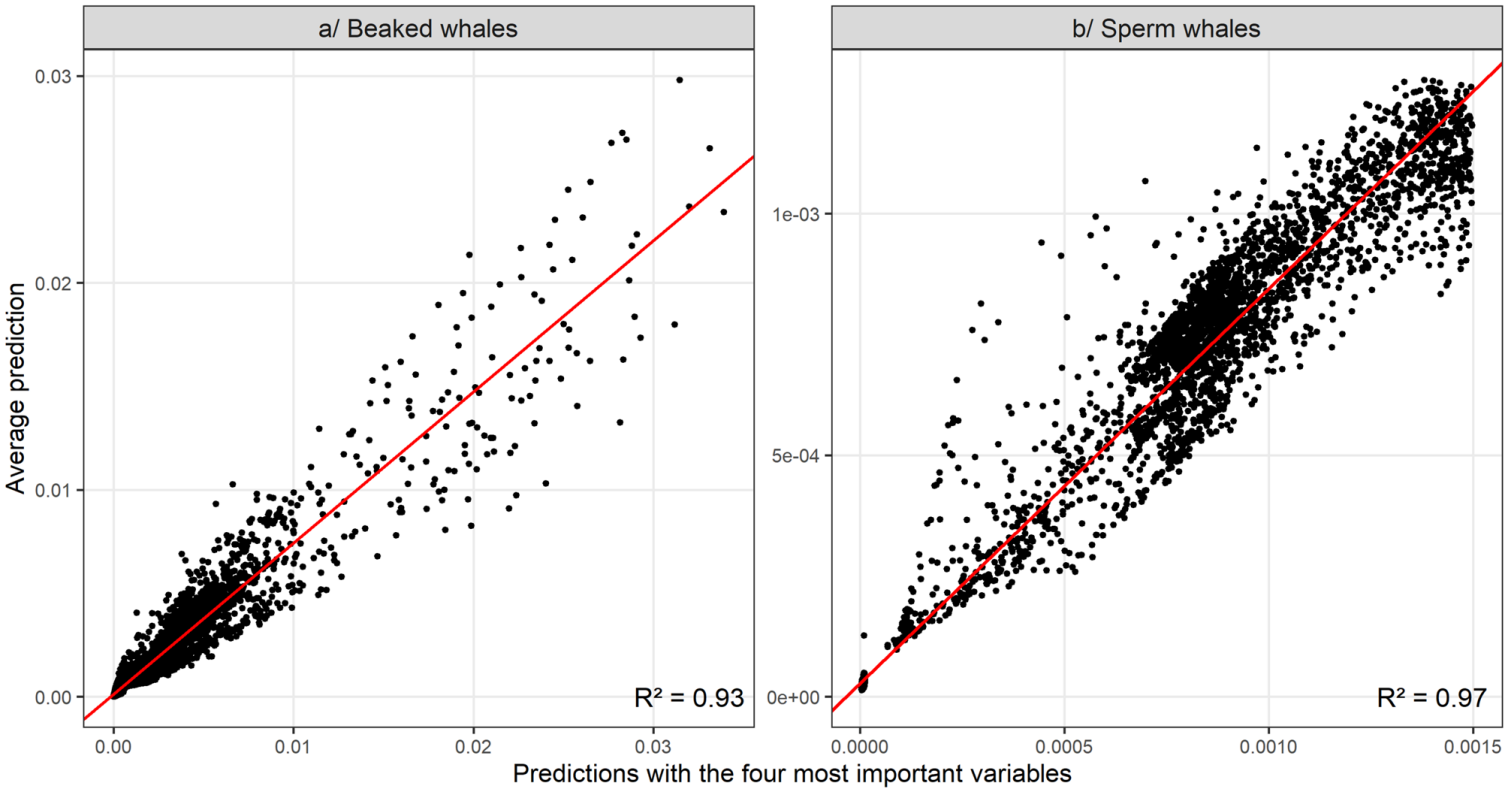
Comparison between the average prediction obtained from the models that explained 80% of the total Akaike weight and the prediction obtained from the model fitted to the four most important variables. If the coefficient of determination (R^2^) is close to 1, predictions are similar and the average prediction of all models can be approximated by the prediction of the model fitted to the four most important variables.

During the model selection process, it turned out that the Akaike weights^32^ of all tested models were much lower than 0.9, probably because some variables were very similar and correlated. Consequently, we tried to approximate the average prediction obtained from all tested models by the prediction obtained from the model that combined the four most important variables for each species (Fig. 1). For both beaked and sperm whales, the coefficient of determination (R^2^) was close to 1 (respectively 0.93 and 0.97) so the predictions were very similar and the average prediction of all models could be approximated by the prediction of the model fitted to the four most important variables.

**Figure 2:**
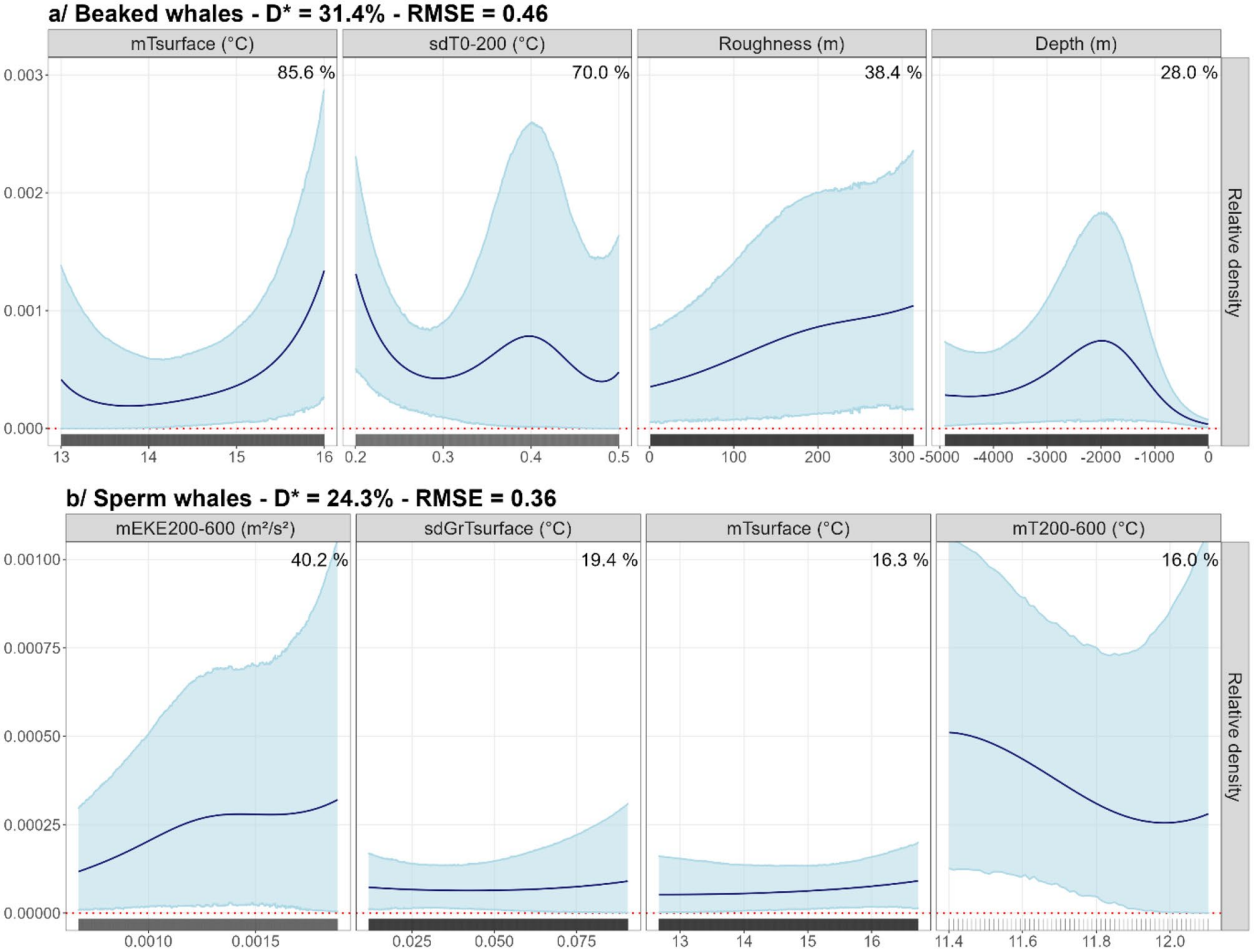
The functional relationships between beaked whale and sperm whale individual densities and the four most important and uncorrelated variables. Solid lines represent the estimated smooth functions and the blue shaded regions show the approximate 95% confidence intervals. The relative density of individuals (individuals per 100 km^2^) is shown on the y-axis, where a zero indicates no effect of the covariate. The black rug plot on the x-axis represents the distribution of the data. The percentages indicate the importance of the variables calculated by summing the Akaike weights of the models in which they were selected. D*: explained deviance; T: temperature; GrT: gradients of temperature; EKE: eddy kinetic energy; m: mean; sd: standard deviation.

The importance of the variables was determined by summing the Akaike weights of the models in which they were selected. For beaked whales, the most important variables were the average temperature at the surface and the standard deviation of the temperature between 0 and 200 m (mT_surface_ and sdT_0–200_) and also static variables (roughness, slope, depth, surface of canyons). For sperm whales, the most important variables were the average temperature and the standard error of the gradients of temperature at the surface (mT_surface_ and sdGrT_surface_) but also variables at depths between 200 and 600 m, the mean and standard deviation of the eddy kinetic energy, the average temperature and the average gradients of temperature (mEKE_200–600_, sdEKE_200–600_, mT_200–600_ and mGrT_200–600_), with mEKE_200–600_ being the most important variable. With the four most important variables, we obtained a unique model that combined the most important environmental variables in the pool of available variables and thus obtained functional relationships with these variables. The final models (Table 1 and Fig. 2) did not necessarily use the four most important variables as some of them were correlated (*e.g.,* roughness and slope).

**Table 1:** Importance of variables ranked by the sum of the Akaike weights of the models in which they were selected.

Beaked whales		Sperm whales	
**mT**_**surface**_	**85.6%**	**mEKE**_**200–600**_	**40.2%**
**sdT**_**0–200**_	**70.0%**	**sdGrT**_**surface**_	**19.4%**
**Roughness**	**38.4%**	**mT**_**surface**_	**16.3%**
Slope	36.1%	**mT**_**200–600**_	**16.0%**
**Depth**	**28.0%**	mGrT_200–600_	15.4%
CanArea	19.9%	sdEKE_200–600_	15.2%

The higher the Akaike weight, the more important is the variable. The variables in bold are the variables selected in the final models used to predict the species distributions. *T* temperature; *GrT* gradients of temperature; *EKE* eddy kinetic energy; *m* mean; *sd* standard deviation.

### Beaked whales

**Figure 3:**
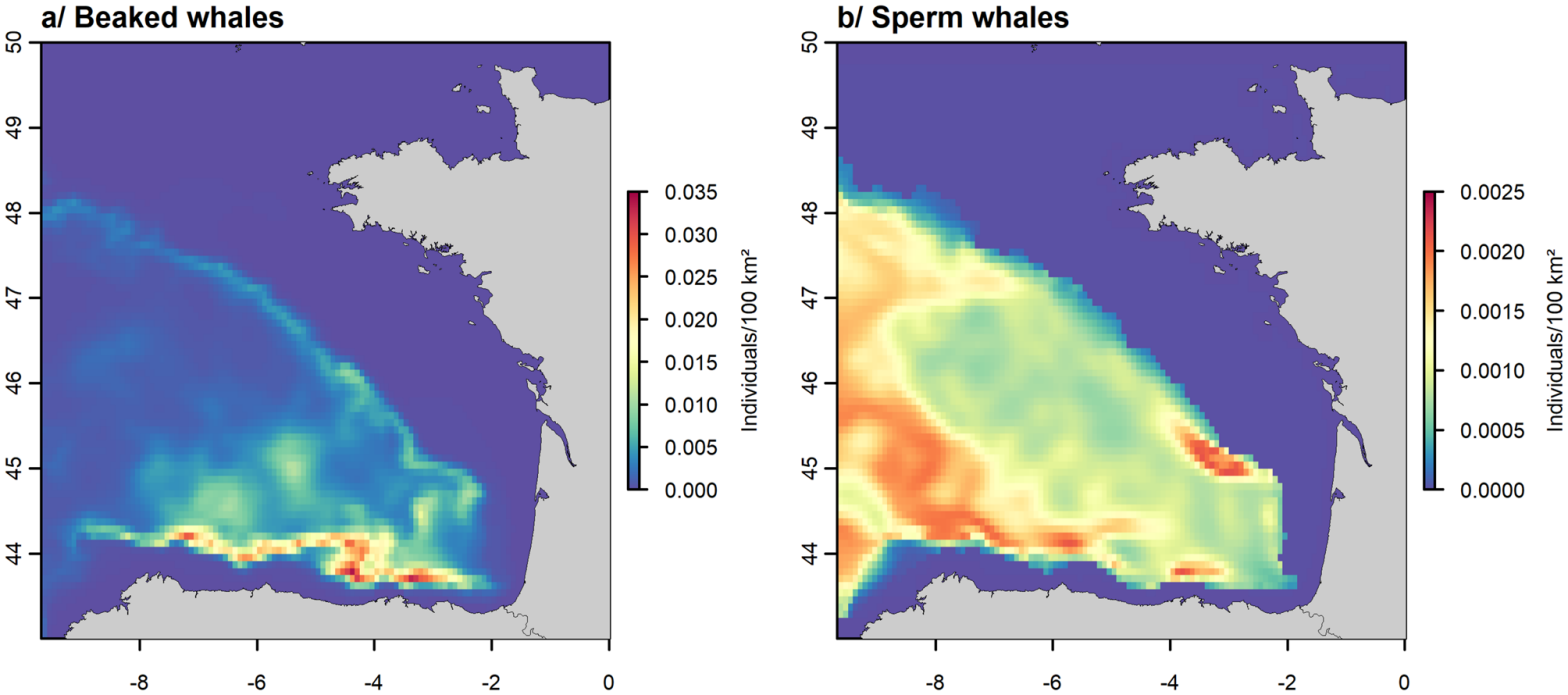
Predicted relative densities of beaked whales (**a**) and sperm whales (**b**) obtained from the models that use the four most important and uncorrelated variables.

The final model of beaked whales explained 31.4% of the deviance and the root mean square error (RMSE) was equal to 0.46. The highest densities were obtained for a mT_surface_ above 15 °C, sdT_0–200_ less than 0.3 °C, a high roughness (> 200 m) and depths around 2000 m (Fig. 2). In the Bay of Biscay, the highest densities of beaked whales were predicted near the continental slope and in the abyssal plain, with maxima predicted in the canyons of the south-eastern Bay of Biscay off the Spanish coast (Capbreton, Torrelavega and Llanes canyons; Fig. 3; map of the Bay of Biscay canyons available in Appendix C). The uncertainties associated with the predictions were low (Appendix D).

### Sperm whale

The explained deviance of the final sperm whale model was 24.3% and the RMSE was equal to 0.36. The highest densities were obtained for mEKE_200–600_ higher than 0.0015 m^2^·s^−2^, sdGrT_surface_ higher than 0.075 °C, mT_surface_ higher than 15 °C and mT_200–600_ less than 11.5 °C (Fig. 2). The highest densities in the Bay of Biscay were predicted in the abyssal plain, near the continental slope and near canyons and seamounts, such as the North Spanish Marginal Trough, the Biscay Seamount and the Cap Ferret and Capbreton canyons, where water movements were very dynamic (Fig. 3; map of the Bay of Biscay canyons available in Appendix C). Although the uncertainties associated with the functional relationships were large, the uncertainty associated with the prediction was fairly low except in the west where the area was less sampled (Appendix D).

## Discussion

For animals that spend most of their time below the surface and feed mostly at great depths, such as beaked and sperm whales^21,22^, using surface environmental variables in SDMs, as commonly done to understand the mechanisms that influence their distribution^5,16,23,24^, may seem ecologically incomplete. The presence of animals at the surface is also probably related to mechanisms at depth. Our objective was to use, in addition to surface environmental variables, variables that depicted the water column to identify which variables were the most important for deep-divers, since we argued that surface variables might not explain well the physical processes occurring at depth that would influence the beaked and sperm whale distributions. Our results highlighted new relationships with the environment allowing to predict the highest densities of beaked whales and sperm whales near the continental slope, near canyons and seamounts and in the abyssal plain of the Bay of Biscay. Interestingly, we identified different responses between beaked whales, for which surface, subsurface and static variables were selected as the most important variables, and sperm whales for which no static variable was selected but only surface and deep-water variables.

It may seem inappropriate to use deep-water variables to explain the distribution of surface sightings but here we assumed that animals were observed at the surface where they were breathing or resting before or after having followed a prey aggregation at depth^12–15^. We expected that variables characterising the water masses at depth would be selected for beaked whales and that static variables would be selected for sperm whales. Brodie et al.^28^, showed that the addition of dynamic subsurface variables might not significantly improve the model predictive performance for species with strong responses to static variables, which would explain our findings for beaked whales. We could also hypothesise that beaked whales might forage on organisms living close to the seabed, explaining why they were more associated to physiographic features around 2000 m depth. By contrast, sperm whales would be less constrained by the presence of the slope or physiographic structures and would prey on organisms that are truly meso-to-bathypelagic; and as a consequence, the dynamic processes in the water column would thus be more important. From the analysis of stomach contents, Spitz et al.^22^ showed that the diets of beaked and sperm whales were different in the Bay of Biscay. Cuvier’s beaked whales fed largely on the cephalopods *Teuthowenia megalops* and *Galiteuthis armata* while the diet of sperm whales was mostly composed of the cephalopods *Histioteuthis bonnellii*. *Teuthowenia megalops* and *Galiteuthis armata* seem to reach greater depths (respectively 1000–2700 m, and 500–2500 m^33^,) than *Histioteuthis bonnellii* for which larger individuals have been recorded to occur between 200 and 800 m in the Atlantic^33^. At-depth dynamic variables should be used in other areas such as the North West Atlantic or the Mediterranean Sea to determine whether the difference in importance of static, surface and deep-water variables between beaked whales and sperm whales would also be observed and would be consistent with whale dietary data available in these areas.

By using deep-water variables with conventional static and surface variables, we highlighted that the highest beaked whale densities were obtained in areas associated with great depths, steep escarpments and high and stable surface temperatures. They appeared to be related to areas of quite high temperatures, to structures present near the 2000 m depth isobath, as shown by Rogan et al.^16^, and to rugged terrain characterised by higher prey richness^34^. The distribution of sperm whales seemed to be more related to dynamic processes at depth with a concentration of animals in dynamic zones where eddies were strong, gradients of temperature were variable, surface temperature was high and temperature at depth was low. This preference for deep waters associated with eddies could be linked to Slope Water Oceanic eDDIES (SWODDIES) structures which are eddies present at 200 m depth that strongly modify the mixing and dispersion of organic matter and thus lead to higher prey concentration^35,36^.

The relationships established in our models generated predicted distributions consistent with field observations and previous studies, in spite of the different variables used. No beaked whales and sperm whales were predicted on the continental shelf where no sightings were recorded, as previously shown by Roberts et al. and Rogan et al.^5,16^, but this does not exclude the occasional presence of these species in this area. The highest densities of beaked and sperm whales were predicted near slope discontinuities where the seafloor is steep and where prey aggregate^37,38^ and near canyons in the south-eastern part of the Bay of Biscay. Sperm whale distribution was slightly more homogeneous and they appeared to be less linked to these structures than beaked whales. These predictions in the Bay of Biscay were consistent with Kiszka et al.^39^, Rogan et al.^16^ and Virgili et al.^31^ studies; there are very few studies carried out on deep-diver habitats in the Bay of Biscay. The concentration of animals around the canyons and shelf edge was consistent with other studies in the Mediterranean Sea or in waters off the western North Atlantic continental shelf edge where higher sighting rates have been recorded in canyon areas^40–43^. The use of smaller segments in the analyses could help refine the understanding of the processes that influence the concentration of these species within the canyons.

Although the explained deviances were relatively high (24.3% and 31.4%) and comparable to other studies (*e.g.* between 30.6 and 33.8% for Rogan et al.^16^ or 34% for Cañadas et al.^23^), the selected models did not entirely explain the distribution of deep-divers as shown by the quite high RMSEs (0.36 and 0.46). This may be due to the methodological choices we made. We chose to create depth classes (in which the variables were averaged) compatible with the water masses identified in the Bay of Biscay but a finer stratification could have been considered, for example every 200 m^36,44^. The predictive power of the beaked whale model might have been reduced by grouping several beaked whale species with different links with the environment or different targeted prey. However, the group of beaked whales was probably mainly composed of Cuvier’s beaked whales^39,45^ so the effect on the model was limited. We chose to fit 'year-round' models because the studied taxa have been reported to show little or no seasonal variation in their habitats (*e.g*.^46,47^). Perhaps we could have increased the predictive power of the models and reduced the uncertainties associated with the predictions by considering only sightings recorded in spring and summer, the most sampled seasons, but we chose to keep the maximum number of sightings to fit the models. In addition, a prior selection of environmental variables, based on our knowledge, had to be made to limit the number of variables to be tested and associated computational burden. We chose to test only three dynamic variables that seemed relevant, temperature, gradients of temperature and eddy kinetic energy, each of them being available for the four depth classes. Other variables could have been considered, such as dissolved oxygen concentration^48^, salinity, mixed layer depth^29^ or isothermal layer depth^28^. Mannocci et al.^48^ have included the depth of the minimum dissolved oxygen concentration because a shallow depth has effects on the physiology of cetacean prey and cause lethargic behaviour which may ease their capture by deep-divers. Brodie et al.^28^ tested two subsurface variables, isothermal layer depth and bulk buoyancy frequency, to model the distribution of four predatory fish species because these variables quantify the structure and stability of the water column. Similarly, Becker et al.^29^ used depth environmental data (temperature, salinity, mixed layer depth) provided by an ocean circulation model, the Regional Ocean Modelling System, to model distribution of cetaceans in the California Current Ecosystem. They both showed that variables obtained from ocean circulation models improved the explanatory power of the distribution models.

The final model, built from the four most important variables was an arbitrary construction from the variables that were the most prevalent in the tested models and which had the highest Akaike weights^49^. We developed a methodology to identify a single model (which only works if predictions are comparable) because it can be difficult, particularly for managers, to interpret the model results to propose conservation measures, especially if the results are an average of several models. Here, the predictions of our final models were very similar or, even equivalent, to the average predictions of all tested models. So, even if our final models were not the only possible ones, the predictions we obtained were quite convincing and the results were easier to interpret than an average of several models. The addition of deep-water variables alone did not entirely explain the distribution of deep-divers but improved the explanatory power of the models and highlighted other processes that could influence their distribution at depth. More direct parameters such as prey distribution data simulated from numerical models could further improve our distribution models^50,51^. However, prey models need refinements to better characterise the prey of deep-diving cetaceans and thus to improve whale distribution models.

The increasing use of deep-water variables or ocean circulation models in SDMs^28,29,52^ can greatly improve the tools available for conservation planning and the management of human activities. The more we are able to understand the mechanisms that influence a species habitat, the more we will be able to predict its distribution and identify areas of animal concentration where efforts must be concentrated to limit the impacts of human activities on the species. By using a finer spatial resolution of the environmental variables and by considering the vertical dimension of the variables, we refined the predictions of the deep-diver distributions compared to our previous study^31^. Our results identified areas of concentration that had not been identified until now in some canyons of the Bay of Biscay (*e.g.* Capbreton, Cap Ferret, Torrelavega and Llanes canyons). As previously mentioned, many improvements can be considered, such as the use of other variables that characterise the water column, but our results confirmed the utility of deep-water variables in SDMs to model the distribution of top predators linked to meso and bathypelagic areas for a better characterising of their habitats. Increasing the use of these variables should be considered to improve the tools available for the planning of human activities, especially for species that would be closely linked to processes at depth. The availability of modelled variables describing deep water-ocean layers should be incorporated into future studies to improve the characterisation of the habitats of top predators.

## Methods

### Study area

The study area encompassed the Bay of Biscay and adjacent waters in the North Atlantic Ocean, from 43 to 50° North and 0 to 10° West. The width of the continental shelf increases from South to North (from 30 to 180 km). The oceanic circulation is characterised by a weak anti-cyclonic circulation in the central zone and becomes cyclonic near the continental shelf. The water column of the Bay of Biscay is divided into four major water masses: (1) between 100 and 600 m, the water column has the characteristics of the central waters of the North Atlantic Ocean; (2) between 600 and 1500 m, Mediterranean waters flow from Gibraltar; (3) between 1500 and 3000 m, there are the deep waters of the Northeast Atlantic and (4) beyond 3000 m the deep Antarctic waters flow northward^36^. Although sperm whales and beaked whales are able to dive beyond 2000 m, many authors [*e.g.*^53–56^] showed that most of the dives made by deep-divers do not exceed 1500–2000 m thus we considered only waters from 0 to 2000 m in this work .

### Data collection and collation

**Figure 4:**
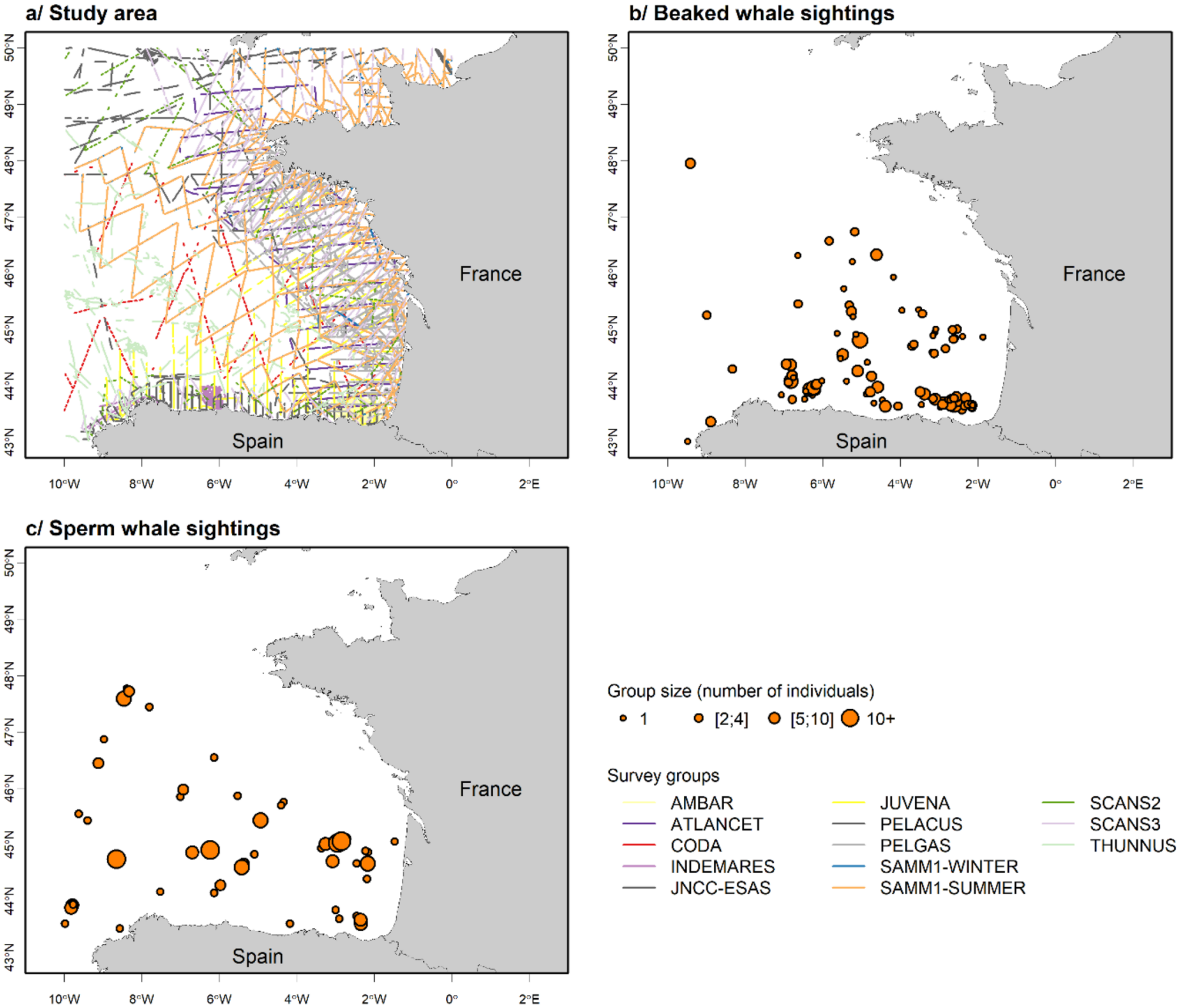
Study area showing assembled survey effort (**a**), along with beaked whale (**b**) and sperm whale (**c**) sightings recorded during all surveys. Surveys were carried out along transects following a line-transect methodology (survey details are provided in Appendix A). Sightings were classified by group sizes with each point representing one group of individuals and point size representing the number of animals in a group. In the analyses, we used the number of individuals to estimate densities of individuals.

In this study, we used a part of the dataset assembled in Virgili et al. (^31^; Fig. 4), we only considered beaked and sperm whale sightings and effort data recorded in the Bay of Biscay, eastern North Atlantic. Visual shipboard and aerial surveys performed by seven independent organisations between 1998 and 2016 were assembled (details of the surveys are listed in Appendix A). A single common dataset was created, aggregating all survey datasets standardised for units and formats. Effort data were linearized and divided into 5 km segments using ArcGIS 10.3^57^ and the Marine Geospatial Ecology Tools software^58^.

Cetacean sightings were recorded following line-transect methodologies that allow Effective Strip Width (ESW) to be estimated from the measurement of the perpendicular distances to the sightings^59^, except for the JNCC-ESAS surveys that used a 300‐m strip‐transect methodology. For each sighting, the number of individuals, the distance from the transect and the conditions of observation were recorded, allowing to build a model which estimated the ESW for beaked whales and sperm whales, depending on observation conditions and survey types (following^31^).

To account for the difficulty to identify them at species level, beaked whale species were pooled into one group including Cuvier’s beaked whales (*Ziphius cavirostris),* mesoplodonts (*Mesoplodon* spp.) and northern bottlenose whales *(Hyperoodon ampullatus)*. Although species were grouped, Kiszka et al.^39^ and Robbins et al.^45^ have shown that the Cuvier’s beaked whale is the most abundant ziphiid in the study area (encounter rates 12 to 16 times higher for Cuvier’s beaked whale than for northern bottlenose whale and Sowerby’s beaked whale (*Mesoplodon bidens*)^45^).

A total of 113 sightings representing 236 individuals of beaked whales and 52 sightings totalling 106 individuals of sperm whales were assembled for the present study (Fig. 4). Aggregated data represented about 150,400 km of on-effort transects (*i.e.* following a transect at specified speed and altitude or height above sea level, with a specified level of visual effort) of which 53% was carried out by boat and the rest by plane (Fig. 4, Table 2).

**Table 2:** Effort performed by platform type and Beaufort sea-state per sector in the North Atlantic Ocean.

	Total survey effort (km and %)	Total aerial effort (km)	Total shipboard effort (km)	Total effort by Beaufort sea-state class (km)				
				0–1	1–2	2–3	3–4	4–7
Study area	150,400	79,100 53%	71,250 47%	35,400 24%	41,400 27%	37,500 25%	23,500 16%	12,600 8%

This table presents the total effort conducted in each sector broken down by platform type and Beaufort sea-state. Beaufort sea-state values reported with decimals in the surveys were rounded up. For the analyses, all segments with Beaufort sea-state > 4 were excluded.

Moran’s and Geary’s indexes were calculated to ensure there was no spatial autocorrelation in the data using the ‘spdep’ R-package^60^.

### Data processing

#### Delimitation of depth classes

**Figure 5:**
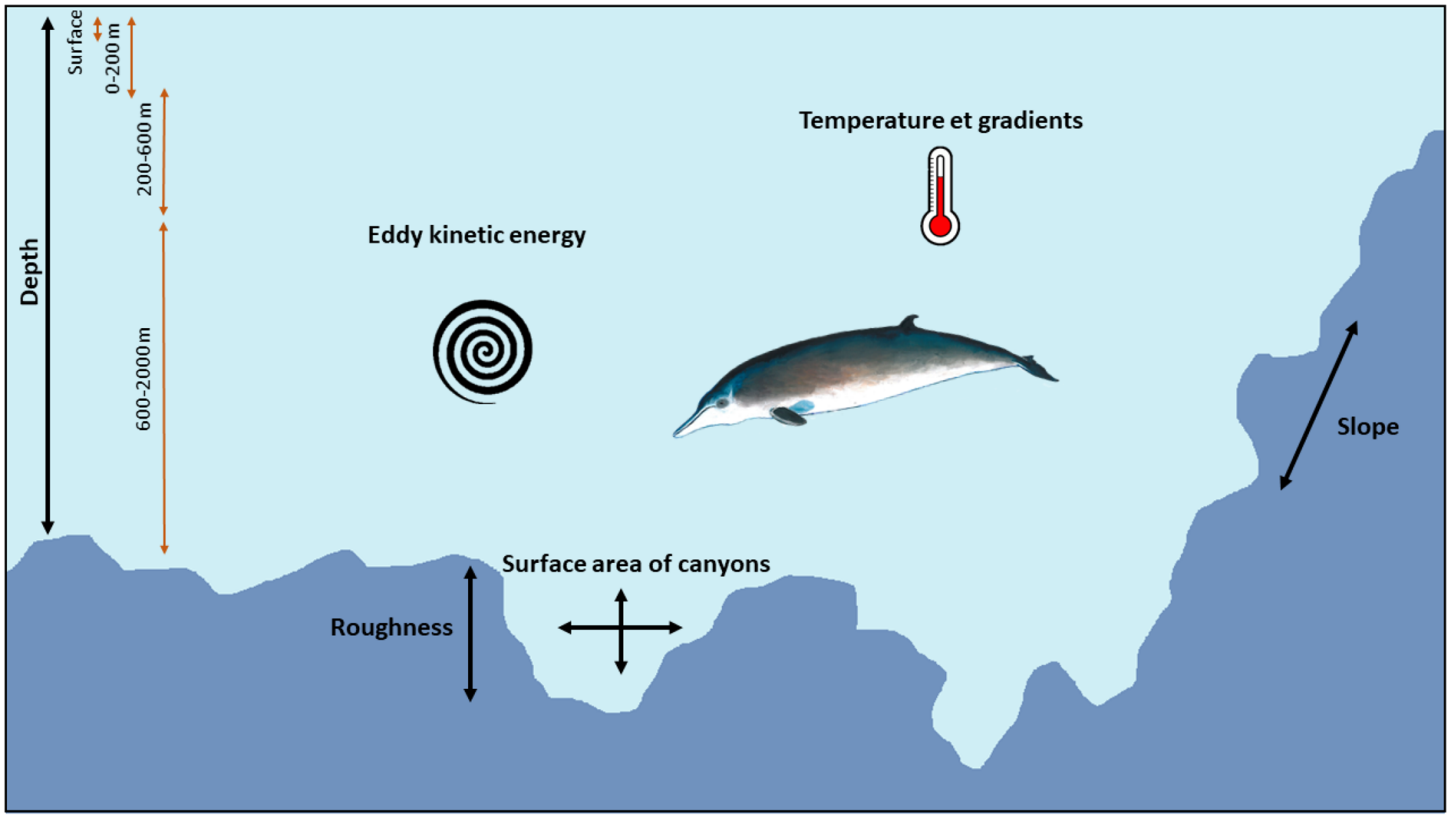
Schematic representation of environmental variables used in habitat-based density models and depth classes. Orange arrows represent the four depth classes (Surface, 0–200 m, 200–600 m and 600–2000 m) and environmental variables are written in black.

To determine whether the presence of deep-divers would be related to surface oceanographic processes or to processes taking place in the water column, four depth classes were delimited according to the water masses reported in the Bay of Biscay (see 4.1) in association with the dive profiles of deep-divers (Fig. 5). Environmental variables were extracted for each of the selected depth classes.

The first class was the “*surface zone*”; the deep-diver distribution may be related to mechanisms at the surface or these mechanisms may influence processes at depth. The second class was the epipelagic zone (named “0–200 m”), extending vertically from 0 to 200 m. We delineated this class between 0 and 200 m because most of beaked whale recovery dives occur between 0 and 200 m^61^ and the upper 200 m of the open ocean correspond to the euphotic zone where primary production is concentrated. Between 200 and 2000 m, we considered two water masses, the central waters of the North Atlantic Ocean between 200 and 600 m (named “200–600 m”) and the Mediterranean waters between 600 and 2000 m (named “600–2000 m”). Although water masses between 600 and 1500 m and between 1500 and 3000 m are different (see 4.1), we considered no changes in the water masses from 1500 to 2000 m and grouped them into one class.

#### Static and oceanographic variables

To model species distributions, it was necessary to extract environmental variables. We considered static and dynamic variables that can affect the distribution of beaked whales and sperm whales (Table 3). Static variables remained stable over time and were independent from depth classes while dynamic variables were extracted in each depth class and varied over time.

**Table 3:** Candidate environmental predictors used for the habitat-based density modelling.

Variables used in the study with abbreviations and units	Original Resolution	Sources	Effects on pelagic ecosystems of potential interest to deep-divers
**Static**			
Depth (m)	15 arc sec	A	Deep-divers feed on squids and fish in the deep-water column
Slope (°)	15 arc sec	A	Associated with currents, high slopes induce prey aggregation or enhanced primary production
Roughness (m) –	15 arc sec	A	A high roughness indicates an important escarpment and a greater richness in prey
Surface of canyons – **CanArea** (km^2^)	15 arc sec	B	Deep-divers are often associated with canyons and seamounts structures
**Dynamic**			
Mean and standard deviation of temperature – **mT**_d1-d2_ and **sdT**_d1-d2_ (°C)	0.083°, daily	C	Variability over time and horizontal gradients of temperatures reveal front locations, potentially associated with prey aggregation or enhanced primary production
Mean and standard deviation of gradients of temperatures – **mGrT**_d1-d2_ and **sdGrT**_d1-d2_ (°C)	0.083°, daily	C	
Mean and standard deviation of eddy kinetic energy – **mEKE**_d1-d2_ and **sdEKE**_1-d2_ (m^2^.s^-2^)	0.083°, daily	C	High EKE relates to the development of eddies and sediment resuspension inducing prey aggregation

A: https://www.gebco.net/; 15 arc-second is approximately equal to 0.004°. B: Harris et al. ^62^. C: Iberian Biscay Irish- Ocean Physics Reanalysis model from Copernicus (https://doi.org/10.48670/moi-00029). All dynamic variables were extracted or computed for each depth class (surface, 0–200 m, 200–600 m and 600–2000 m). Abbreviations used in the following article are defined here in bold, d1-d2 refers to the depth classes e.g. 200–600 means between 200 and 600 m. In the analyses, all variables were resampled or used at a 0.083° spatial resolution.

For static variables, we extracted the depth at a 15 arc second resolution (≈ 500 m; https://www.gebco.net/) and then computed the slope (inclination of the seafloor) and roughness (difference between the maximum and minimum depth of the pixels surrounding the central pixel) with the function ‘terrain’ from the R package ‘raster’^63^. We also extracted the surface area of canyons listed in the study area because deep‐divers are often associated with canyon structures (^62^; www.bluehabitats.org). All static variables were resampled at a 0.083° resolution to match the resolution of the dynamic variables.

For dynamic variables, we extracted monthly water temperatures and current vectors (U and V) for each depth class (surface, 0–200 m, 200–600 m and 600–2000 m) directly from the numerical model “Iberian Biscay Irish Ocean Reanalysis” of Copernicus (itself based on the NEMO v3.6 ocean general circulation model; https://doi.org/10.48670/moi-00029). From these variables, we computed spatial gradients of temperatures and EKE (0.5*(U^2^ + V^2^)). Gradients of temperatures were calculated as the difference between the minimum and maximum temperature values found in the eight pixels surrounding any given pixel of the grid (function ‘detectFronts’ from the R package ‘grec’^64^). Climatological variables were computed by calculating the means and standard deviations over the study period for each variable of each depth class (Appendix B).

### Habitat-based density modelling

To model the distribution of beaked whales and sperm whales, we fitted GAMs^30^ with a Tweedie distribution to account for over-dispersion in the cetacean count data^65^ with the ‘mgcv’ R-package^66^. GAMs extend generalised linear models to allow for smooth, nonlinear functions of predictor variables to be determined by observed data rather than by strict parametric relationships^30^. The mean number of individuals per segment was linked to the additive predictors with a log-function with four degrees of freedom. An offset equal to segment length multiplied by twice the ESW, or twice the 300 m-strip for JNCC-ESAS surveys, was included (^67^; refer to Virgili et al.^31^ for the ESW estimation); ESWs were calculated for each combination of platform, class of Beaufort sea-state and class of observation height. We removed combinations of variables with Pearson correlation coefficients higher than |0.5| and tested all models with combinations of one to four variables to avoid excessive complexity^68^.

**Figure 6:**
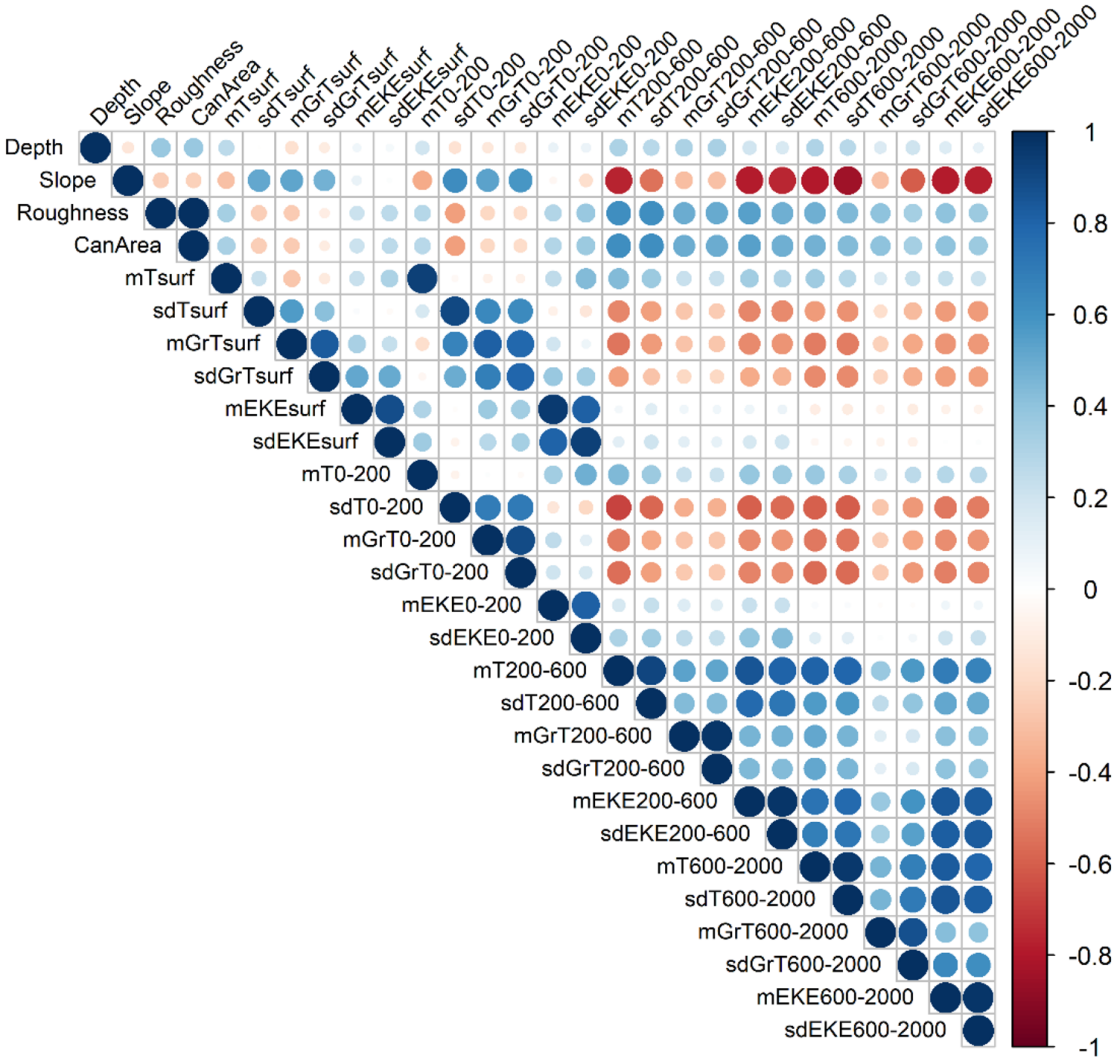
Correlation matrix of environmental variables. This matrix was calculated using the Pearson coefficient; the larger and darker the circle, the higher the correlation between the two environmental variables; variables are considered correlated for values below − 0.5 and above 0.5. CanArea: surface of canyons; T: temperature, GrT: gradients of temperature; EKE: eddy kinetic energy; m: mean; sd: standard deviation; surf: surface; 0–200: 0–200 m; 200–600: 200–600 m; 600–2000: 600-2000 m.

To assess the correlation between the variables we created a correlation matrix using the R package ‘corrplot’ (^69^; Fig. 6). Many variables with depths greater than 200 m were correlated with each other but also with surface variables (depth < 200 m) and with static variables such as slope, roughness and surface of canyons; showing the importance of considering correlations in the model selection.

The Akaike information criterion (AIC, the lower the better^32^) and Akaike weight (w; ‘akaike.weights’ function from ‘qpcR’ R package^70^;) were used for model selection. The Akaike weight of a model is the probability that this model is the best among all tested models^32,71^. If the Akaike weight of a model is high (w > 0.9), it can certainly be identified as the best model and inference can be made from this model alone^32,49^. In contrast, if w < 0.9, a model averaging is recommended, which consists in producing parameter estimates from the weighted average of several models and not from a single model^49^. If w < 0.9, some variables are probably very similar and correlated. In this case, it is not possible to choose a best model among all tested models since the models are equivalent and they must all be integrated to produce an average prediction of species distribution. This can be cumbersome in terms of calculations and difficult to interpret, so if possible, it is more suitable to obtain a single model.

To be able to identify this single suitable model, we aimed to approximate the average prediction obtained from the models by the prediction obtained from the model that combined the four most important variables. Following Symonds & Moussalli^49^, we determined the importance of each variable by summing the Akaike weights of the models in which the variable was selected and ranked all variables. A model using the first four variables was then fitted while ensuring a non-correlation of the variables (if variables were correlated, the next uncorrelated ranked variable was chosen). A prediction of relative densities (in number of animals per pixel) was produced from this model at a 0.083° resolution and compared to the average prediction obtained from the models that explained 80% of the total Akaike weight. We considered only the models that explained 80% of the Akaike weight because beyond this threshold, the models were negligible (very low explained deviances and very high AICs). If the coefficient of determination (R^2^) of the regression line established between the values of the average prediction and the values of the prediction obtained from the four most important variables was close to 1, predictions were similar and the average prediction of all models could be approximated by the prediction of the model fitted to the four most important variables. The four variables could therefore be used to obtain functional relationships and to predict the relative densities of beaked whales and sperm whales in the Bay of Biscay. If not, all models had to be considered to predict the species densities.

There were not enough data to fit a model by month or by season (the number of individuals in winter was too low) so we fitted models to all data of beaked whales and sperm whales and obtained climatological predictions maps for all seasons combined in the 1998–2016 period, although most of sighting data were collected in summer and the prediction maps most likely reflected the summer species distribution. The uncertainties associated with the predictions were also estimated as the standard deviation associated with the predicted relative densities; high values indicated high errors associated with density estimates. However, it should be noted that the uncertainty associated with the model prediction with the four most important variables was certainly lower than the uncertainty associated with the mean prediction of all models and it was therefore underestimated.

Finally, the model fit of each model was assessed thanks to the percentage of explained deviance^30,72^, the Root Mean Squared Error (RMSE) which measured the prediction errors and the model accuracy (the lower, the better^73^; ‘qpcR’ R-package^70^) and a visual inspection of predicted and observed distributions^74^.

## Supplementary Information

Supplementary Information.

## Data Availability

All sighting and effort data used in this study are available in the GitHub: https://github.com/avirgi01/DeepVariables.git. All data providers can be contacted via the email addresses provided in the data files.
