# Supplementary material for: Deep ocean drivers better explain habitat preferences of sperm whales Physeter macrocephalus than beaked whales in the Bay of Biscay
Source: Sci Rep. 2022 Jun 10;12:9620. doi: 10.1038/s41598-022-13546-x (PMC9187681; doi:10.1038/s41598-022-13546-x)

Appendix A: details of surveys used in the analyses. Total effort represents the total length of transects of each survey (without removing the transects with a Beaufort sea-state > 4). Bob: Bay of Biscay; NE-ATL: Northeast Atlantic Ocean; Org: organisation; Nb sight (ind): number of sightings and number of individuals; BW: beaked whales; SW: sperm whales; Ref: references.

**Survey name (Fig.1)**	**Org**	**Plateform type**	**Region**	**Years**	**Total effort (km)**	**Nb sight (ind) BW**	**Nb sight (ind) SW**	**Ref**
AMBAR	AMBAR	Ship	South east of the BoB	2004-2005	5,100	20 (49)	1 (1)	[1-3]
ATLANCET	Pelagis	Plane	Bob	2002	3,800	5 (10)	1 (1)	[4]
CODA	SMRU	Ship	NE-ATL	2007	2,600	17 (36)	18 (39)	[5]
INDEMARES	CEMMA	Ship	NE-ATL	2009-2011	3,900	11 (24)	0 (0)	[6]
JNCC-ESAS	JNCC	Ship	NE-ATL	1998-2005 ; 2008	6,700	0 (0)	0 (0)	[7]
JUVENA	AZTI	Ship	Bob	2012-2015	8,900	8 (19)	5 (7)	[8]
PELACUS	Instituto Español de Oceanografía (IEO)	Ship	North and NW Spanish shelf waters	2007-2012	6,900	2 (2)	1 (1)	[9]
PELGAS	Pelagis	Ship	Bob	2007-2013	35,000	6 (10)	14 (34)	[4]
SAMM	Pelagis	Plane	Bob	2011-2012	57,500	22 (44)	8 (12)	[10]
SCANS 2	SMRU	Ship	NE-ATL	2005	3,800	9 (17)	0 (0)	[5]
SCANS 3	SMRU	Plane	NE-ATL	2016	9,900	9 (16)	0 (0)	[11]
THUNNUS	CEMMA	Ship	Bob	2008-2010	6,300	4 (9)	4 (11)	[12]
**TOTAL**					**150,400**			

References

1. Vázquez, A., Ruiz, L., Maestre, Z., Ruiz-Gondra, J., Ruiz-Guijarro, J., Benedicto, L., … Goenaga I. Landbased sightings from the Basque Country coast (northeast Spain). 17th Annual Conference of the European Cetacean Society, Las Palmas de Gran Canaria, Canary Islands (Spain). (2003).

2. Vázquez, J.A., Cermeño, P., Williams, A., Martin, C., Lazkano, O., Ruiz, L., … Guzman, I. Identifying areas of special interest for Cuvier's beaked whale (Ziphius cavirostris) in the southern part of the Bay of Biscay. In Abstracts, 18th Annual Conference of the European Cetacean Society, Kolmårdon, Sweden. (2004).

3. Vázquez, J.A., Guzmán, I. Lazkano, O., & Olondo, M. Encounter rates of small cetaceans, pilot whales and Ziphiidae in coastal waters of Basque Country (Southern Bay of Biscay). 19th Annual Conference of the European Cetacean Society, La Rochelle, France. (2005).

4. Certain, G., Ridoux, V., Van Canneyt, O., & Bretagnolle, V. Delphinid spatial distribution and abundance estimates over the shelf of the Bay of Biscay. *ICES Journal of Marine Science* **65**(4): 656-666. (2008).

5. Rogan, E., Cañadas, A., Macleod, K., Santos, M.B., Mikkelsen, B., Uriarte, A., … Hammond, P.S. Distribution abundance and habitat use of deep diving cetaceans in the North-East Atlantic. *Deep Sea Research Part II: Topical Studies in Oceanography* **141**: 8-19. (2017).

6. López, A., & Martínez-Cedeira, J. Final report of the project "LIFE 07/NAT/E/000732 INDEMARES". Unpublished technical report. CEMMA. 305 pp. (2012).

7. Reid, J.B., Evans, P.G., & Northridge, S.P. Atlas of cetacean distribution in north-west European waters. Joint Nature Conservation Committee. (2003).

8. Boyra, G., Martinez, U., Cotano, U., Santos, M., Irigoien, X. & Uriarte, A. Acoustic surveys for juvenile anchovy in the Bay of Biscay: abundance estimate as an indicator of the next year's recruitment and spatial distribution patterns. *ICES Journal of Marine Science* **70**:1354–1368. (2013).

9. Santos, M.B., González-Quirós, R., Riveiro, I., Iglesias, M. Louzao, M., & Pierce, G.J. Characterization of the pelagic fish community of the North Western and Northern Spanish shelf waters. *Journal of Fish Biology* **83**(4): 716-738. (2013).

10. Lambert, C., Pettex, E., Dorémus, G., Laran, S., Stephan, E., Van Canneyt, O., & Ridoux, V. How does ocean seasonality drive habitat preferences of highly mobile top predators? Part II: the eastern North- Atlantic. *Deep Sea Research Part II: Topical Studies in Oceanography* **141**: 133-154. (2017).

11. Hammond, P. S., Lacey, C., Gilles, A., Viquerat, S., Börjesson, P., Herr, H., ... & Øien, N. Estimates of cetacean abundance in European Atlantic waters in summer 2016 from the SCANS-III aerial and shipboard surveys. Wageningen Marine Research. (2017).

12. Martínez-Cedeira, J., & López, A. Final Report Thunnus 2007-2010 Surveys. Unpublished technical report. CEMMA. 87 pp. (2010).

Appendix B: average conditions of the static and deep-water variables over the entire period (from 1998 to 2016).

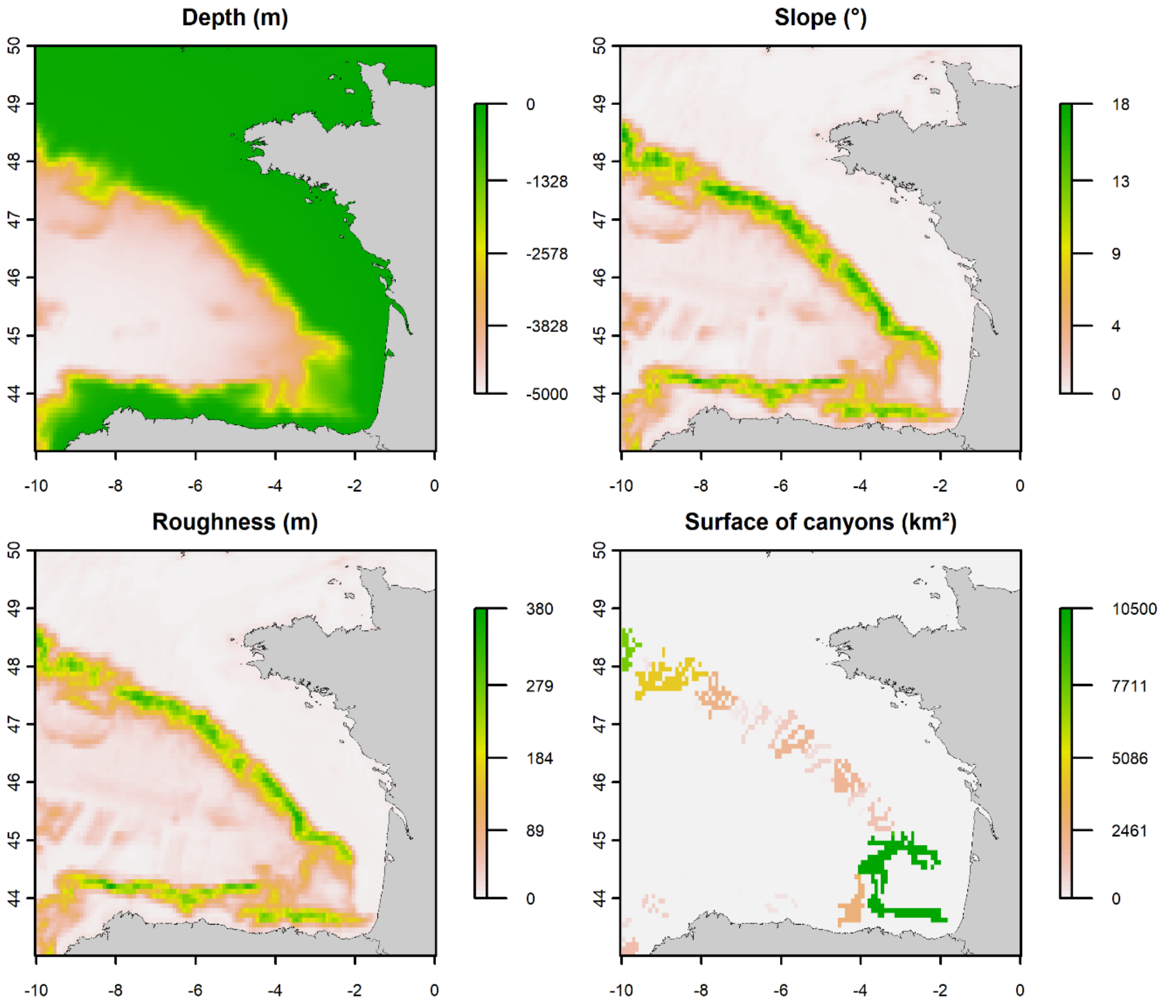

Figure 1. Static variables.

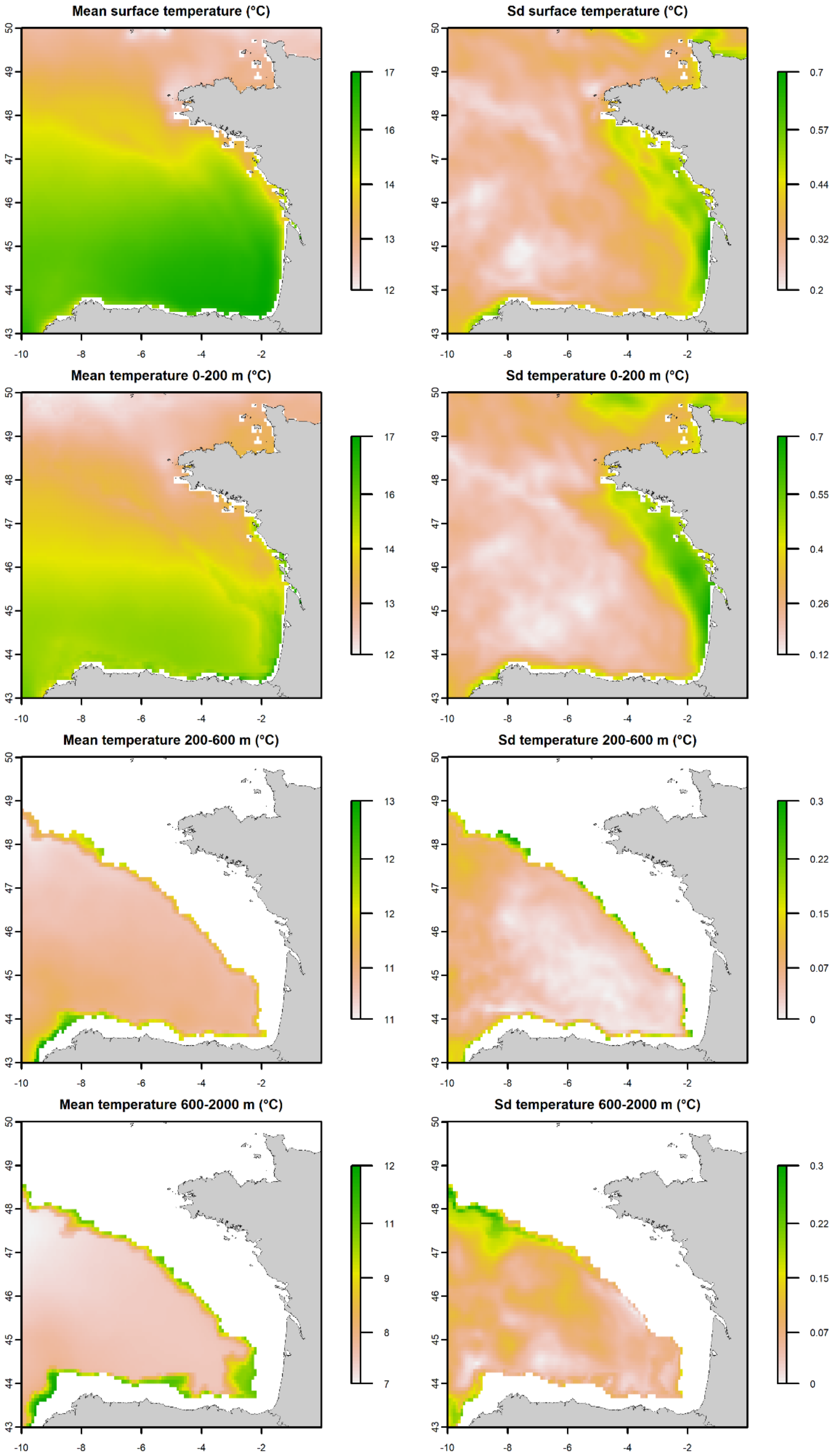

Figure 2. Average conditions of the temperature over the entire period (from 1998 to 2016) for each depth class. Sd: standard deviation.

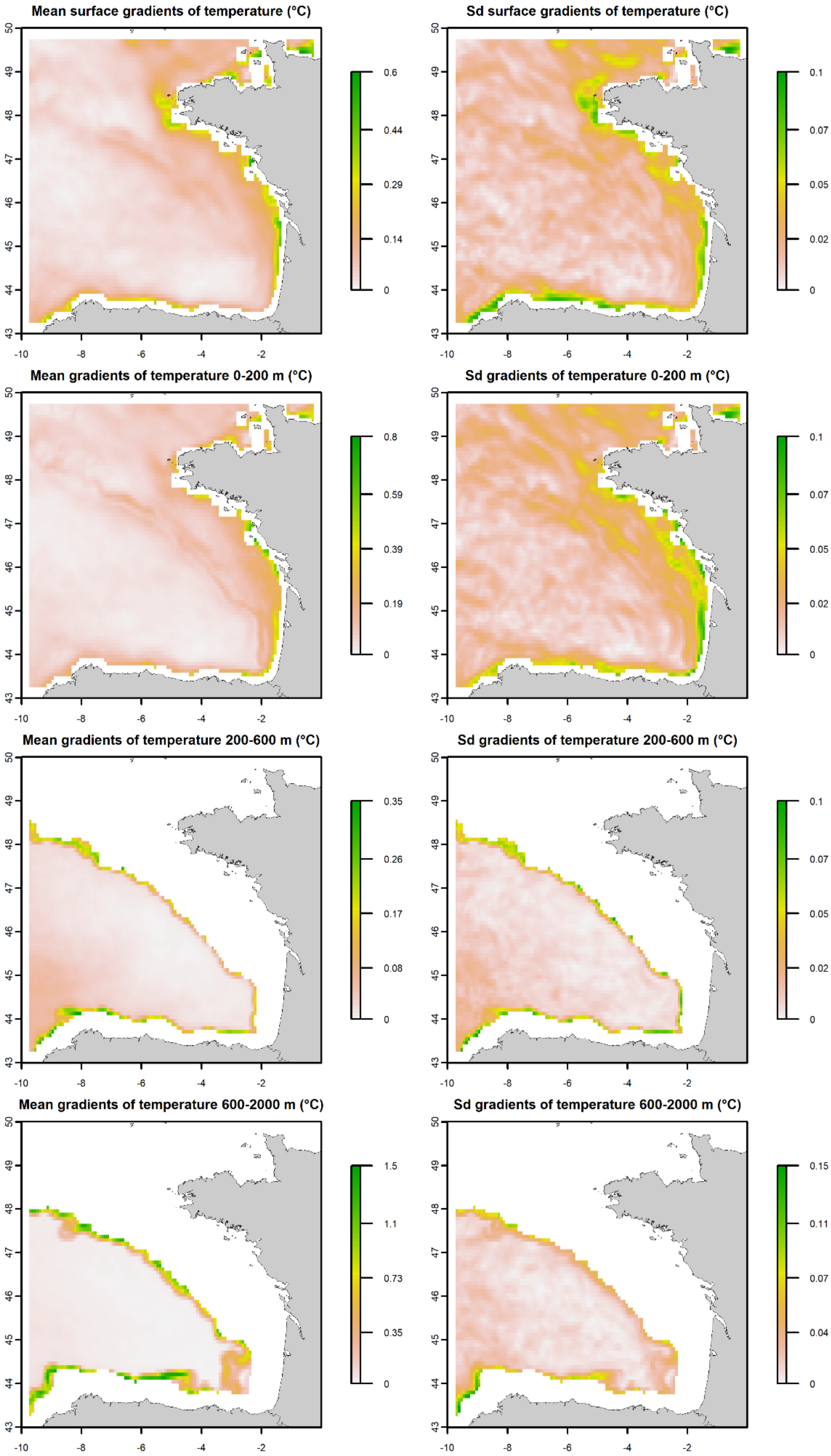

Figure 3. Average conditions of the gradients of temperature over the entire period (from 1998 to 2016) for each depth class. Sd: standard deviation.

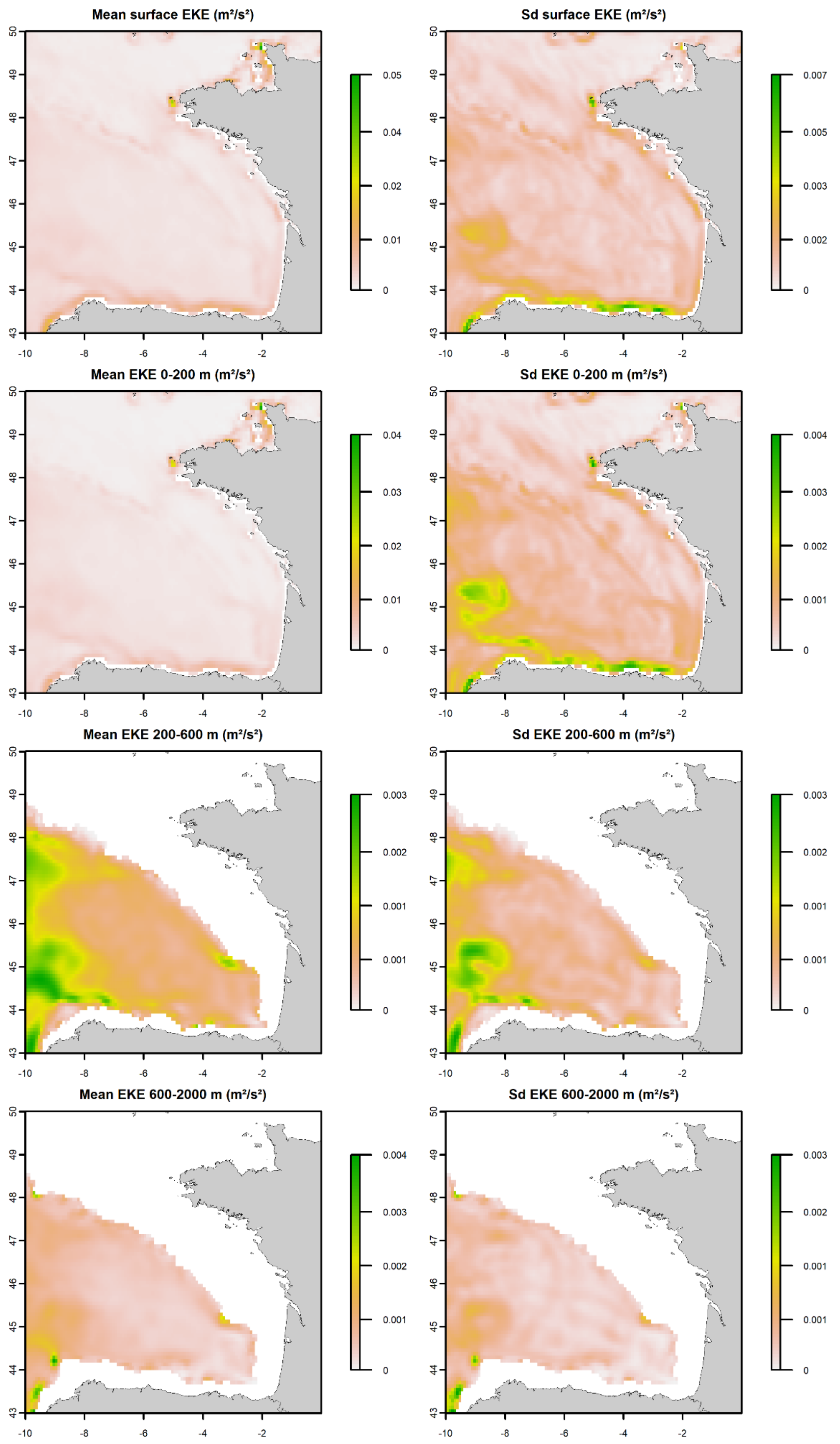

Figure 4. Average conditions of the eddy kinetic energy (EKE) over the entire period (from 1998 to 2016) for each depth class. Sd: standard deviation.

Appendix C: bathymetry of the Celtic Sea and the Bay of Biscay (Eric Gaba (Sting - fr:Sting), CC BY-SA 3.0 <https://creativecommons.org/licenses/by-sa/3.0>, via Wikimedia Commons; downloaded from https://upload.wikimedia.org/wikipedia/commons/c/c1/Celtic_Sea_and_Bay_of_Biscay_bathymetric_map-en.svg).

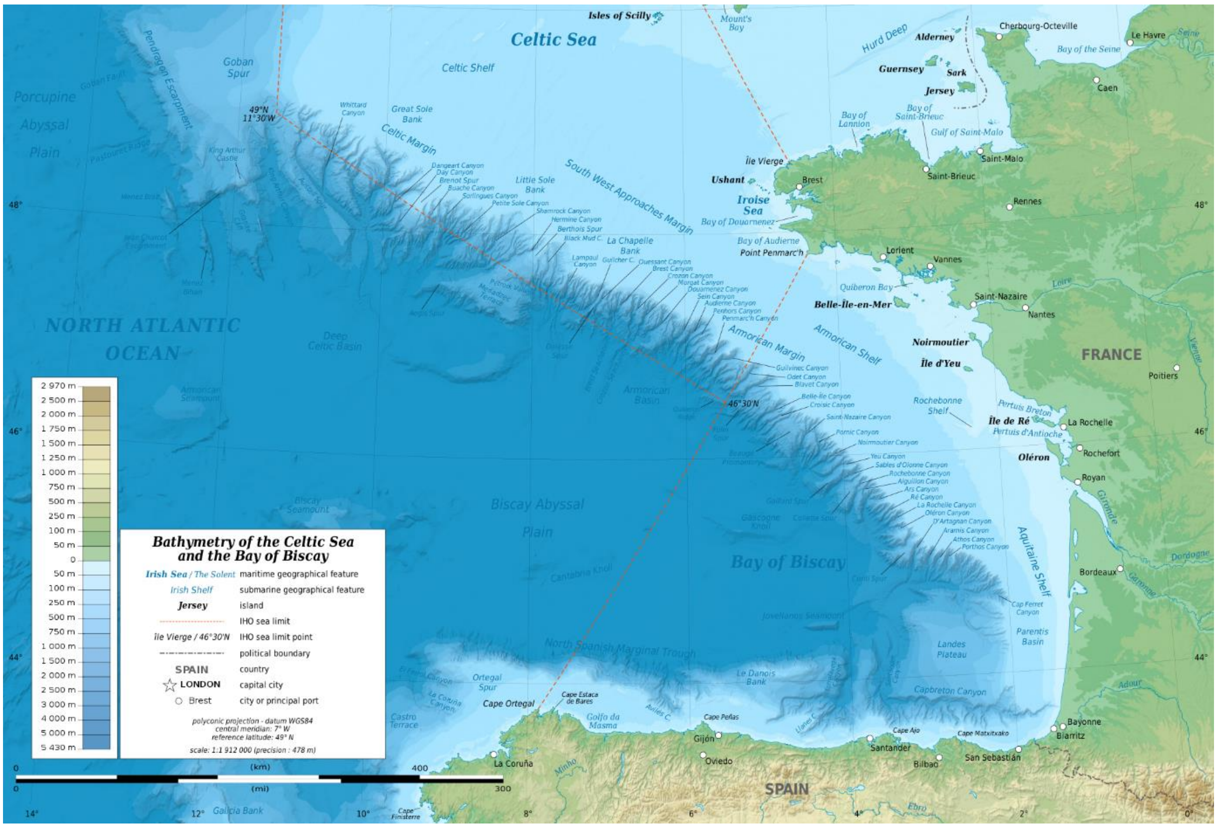

Appendix D: uncertainty maps representing the standard error associated with the predicted relative density of (a) beaked and (b) sperm whales.

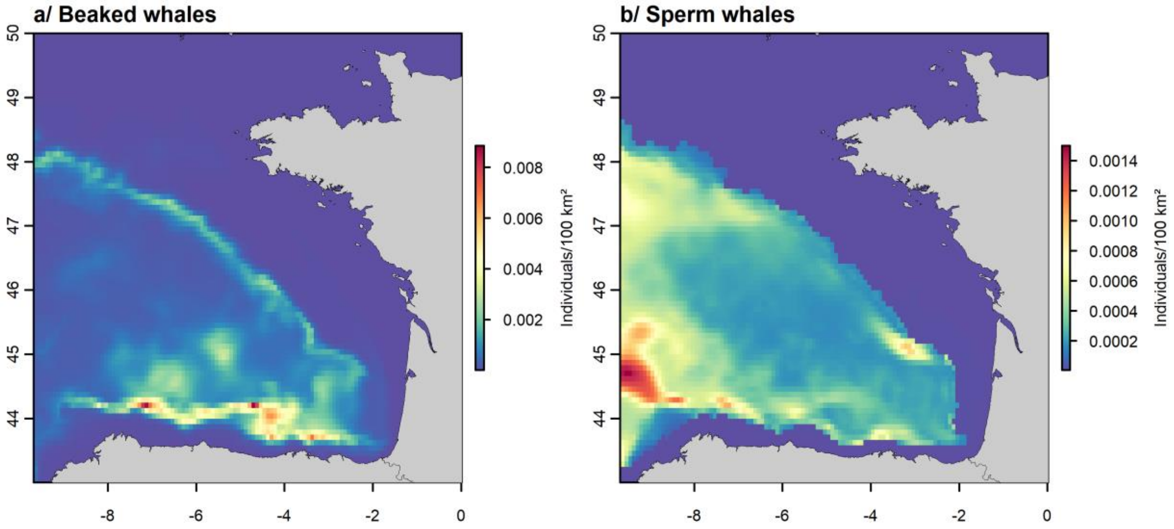

Supplement: Supplementary file 1 — Supplementary Information. [file 41598_2022_13546_MOESM1_ESM.pdf]
